# Evolution, Transmission, and Pathogenicity of High Pathogenicity Avian Influenza Virus A (H5N8) Clade 2.3.4.4, South Korea, 2014–2016

**DOI:** 10.3389/fvets.2022.906944

**Published:** 2022-06-21

**Authors:** Yoon-Gi Baek, Yu-Na Lee, Yu-Ri Park, David H. Chung, Jung-Hoon Kwon, Young-Jae Si, Gyeong-Beom Heo, Youn-Jeong Lee, Dong-Hun Lee, Eun-Kyoung Lee

**Affiliations:** ^1^Avian Influenza Research & Diagnostic Division, Animal and Plant Quarantine Agency, Gimcheon-si, South Korea; ^2^Department of Pathobiology and Veterinary Science, University of Connecticut, Storrs, CT, United States; ^3^College of Veterinary Medicine, Kyungpook National University, Daegu, South Korea; ^4^College of Veterinary Medicine, Konkuk University, Seoul, South Korea

**Keywords:** HPAI, H5N8, pathogenesis, selection pressure, phylodynamic analysis

## Abstract

During 2014–2016, clade 2.3.4.4 H5N8 high pathogenicity avian influenza virus (HPAIV) caused the largest known avian influenza epidemic in South Korea. Based on data from earlier H5N8 outbreaks, primitive H5N8 virus in South Korea was classified into five subgroups: C1, C2, C3, C4, and C5. The present study investigated the pathogenic and molecular epidemiologic characteristics of H5N8 viruses obtained from 388 cases of poultry farms and 85 cases of wild bird infections in South Korea during 2014–2016. Representative viruses of subgroups C1, C2, and C4 showed significant pathobiological differences in specific pathogen-free (SPF) chickens, with the H1731 (C1) virus showing substantially lower infectivity, transmissibility, and pathogenicity than the H2102 (C2) and H1924 (C4) viruses. Full genome sequence analysis showed the number of mutations that significantly increased in domestic duck-origin H5N8 HPAIVs compared to the viruses from gallinaceous poultry. These differences may have been due to the long-term circulation of viruses in domestic duck farms. The same mutations, at positions 219 and 757 of PB1, independently evolving in the C0, C1, and C2 subgroups may have been positively selected, resulting in convergent evolution at the amino acid level. Bayesian discrete trait phylodynamic analysis (DTA) indicated multiple introductions of H5N8 HPAIV from wild birds into domestic poultry in various regions in South Korea. Following initial viral introduction into domestic duck farms in the western part of Korea, domestic ducks played a major role in viral transmission and maintenance. These findings highlight the need for continued genomic surveillance and pathobiological characterization of HPAIV in birds. Enhanced biosecurity in poultry farms should be implemented to prevent the introduction, maintenance, and spread of HPAIV.

## Introduction

The H5 subtype of high pathogenicity avian influenza viruses (HPAIVs) belonging to the Goose/Guangdong (Gs/Gd) lineage have been circulating since 1996 and evolving into genetically diverse clades and subclades (1–3). The H5N8 subtype of Gs/Gd HPAIV belonging to the subclade 2.3.4.4 was initially identified in domestic ducks from eastern China in 2010 (4) and novel reassortant H5N8 HPAIVs were detected in live poultry markets in eastern China in late 2013. Subsequently, reassortant H5N8 HPAIVs were introduced into South Korea and Japan in early 2014 (5–7). In late 2014, several countries in Europe and North America experienced an invasion of HPAI H5Nx viruses (8–11). The transcontinental spread of these viruses was associated with dissemination in multiple directions by migratory wild birds (12).

In South Korea, four outbreaks of H5N1 viruses have been recorded, in 2003–2004 (clade 2.5), 2006–2007 (clade 2.2), 2008 (clade 2.3.2), and 2010–2011 (clade 2.3.2.1), with the lengths of these outbreaks ranging from 42 (2008) to 139 (2010–2011) days (13). The fifth HPAI outbreak started in January 2014 and lasted for 28 months (14). During this outbreak, South Korea experienced four waves of H5N8 outbreaks with 393 cases of poultry farms and 58 cases of wild bird infections. Moreover, two genetically distinct groups of clade 2.3.4.4 H5N8 viruses were identified: Buan2-like and Gochang1-like. The Buan2-like H5N8 viruses predominated during the epidemic and further diverged into five distinct subgroups: C1, C2, C3, C4, and C5 (8, 14). After the first wave, from January to July 2014, subgroups C1 and C5 were detected mainly in poultry farms in South Korea, whereas the other three subgroups were detected in wild birds and spread over long distances via wild birds including subgroups C2 in South Korea and Japan, C3 in North America and Japan, and C4 in Europe, Japan, and South Korea (8–11). The C2 and C4 subgroups were maintained in wild birds and re-introduced into South Korea during the fall migration season of wild birds in 2014.

Little is currently understood about the evolution and spread of H5N8 HPAIV during the 2014–2016 outbreak in South Korea. Previous studies investigating the origin and transmission of H5N8 HPAIV in South Korea included limited datasets, such as partial genome sequences of early isolates or selected viruses (8, 14, 15). The present study analyzed the complete genome sequences of H5N8 HPAIVs identified in 388 (of 393 total cases) poultry farms and 53 (of 58 total cases) wild bird cases during the 2014–2016 outbreak in South Korea. The pathogenic and molecular epidemiologic characteristics of the viruses were determined to identify the underlying causes of the prolonged outbreak and to reconstruct the evolutionary and transmission dynamics of H5N8 HPAIVs in South Korea.

## Materials and Methods

### Samples

Fecal samples, bird carcasses, and oropharyngeal and cloacal swabs were collected from poultry farms and wild bird habitats in South Korea through active and passive surveillance from January 2014 to June 2016. Viruses were isolated by inoculating samples into 9–11-day-old specific-pathogen-free (SPF) embryonated chicken eggs after incubation for 4 days at 37°C.

### Genome Sequencing

Viral RNA was extracted from the infected allantoic fluids of SPF chicken eggs using Maxwell® RSC simplyRNA Tissue Kits (Promega, Madison, WI, USA). Complementary DNA was synthesized by reverse transcription reaction using Superscript III First-Strand Synthesis System (Invitrogen, Carlsbad, CA, USA) and eight segments of each virus were amplified by PCR using AccuPrime Pfx DNA Polymerase (Invitrogen, Carlsbad, CA, USA), as described (16). The complete genome sequences of 441 H5N8 HPAIVs isolated from 388 domestic poultry farms and 53 wild bird habitats were determined by next-generation sequencing with the Illumina Miseq platform using the Nextera DNA Flex Library Prep Kit (Illumina, San Diego, CA, USA) according to the manufacturer's instructions. Data were analyzed by CLC Genomics Workbench (Qiagen, Valencia, CA, USA). Nucleotide sequences were deposited under accession nos. EPI_ISL_157609-EPI_ISL_410213.

### Genetic Subgrouping by Maximum-Likelihood Phylogenetic Analysis

The complete genomes of the 441 H5N8 HPAIVs sequenced in this study were subjected to phylogenetic analyses, along with 32 previously determined whole genome sequences of clade 2.3.4.4A H5N8 HPAIV identified in wild waterfowl in South Korea that had been deposited in the GISAID EpiFlu database (http://www.gisaid.org). Furthermore, our data set was added 20 sequences of the H5N8 viruses isolated from poultry and wild waterfowl in Japan. Maximum-likelihood (ML) phylogenies of the 493 concatenated complete genome sequences were generated using the Randomized Axelerated Maximum Likelihood (RAxML 8.2.12) (17) method, involving the general time reversible model of nucleotide substitution and the Gamma model of among-site rate heterogeneity model with 1,000 bootstrap iterations. A genetic subgroup was defined as a monophyletic cluster of sequences with high bootstrap support (>70%).

### Animal Experiments

The objective of this study was to evaluate the infectivity, transmissibility, and pathogenicity of the first detected viruses (index virus) of three subgroups (C1, C2, and C4 except for C5 with only four cases) of H5N8 HPAIVs in chickens. Viruses evaluated in chickens included A/broiler duck/Korea/H1731/2014 (subgroup C1, abbreviated H1731), A/mallard/Korea/H2102/2015 (subgroup C2, abbreviated H2102), and A/mallard/Korea/H1924/2014 (subgroup C4, abbreviated H1924). To determine the mean bird lethal dose (BLD_50_), five 4-week-old SPF white leghorn chickens each were inoculated intranasally with serial 10-fold dilutions (10^3^ to 10^6^ mean egg infectious dose [EID_50_]) of each virus. Viral transmissibility was evaluated by housing three virus-naïve chickens with the chickens challenged with 10^6^ EID_50_/0.1 ml of each virus 8 h after infection. The chickens were monitored daily for clinical signs for 14 days. Oropharyngeal and cloacal swabs were collected at 1–7, 10, and 14 days post-infection (dpi). Serum was collected from surviving chickens at 14 dpi to determine seroconversion by hemagglutination inhibition test (HI) (18). The tissue tropism and dissemination of the viruses were evaluated by intranasally inoculating three chickens with 10^6^ EID_50_/0.1 mL of each virus. These chickens were necropsied at 3 dpi and 12 organs (trachea, thymus, liver, pectoral major muscle, spleen, proventriculus, pancreas, cecal tonsil, lung, kidney, heart, and brain) were collected for viral titration. Virus titers were measured in DF-1 cells incubated with 10% tissue homogenates. Experiments in animals were reviewed and approved by the Institutional Animal Care and Use Committee of the Animal and Plant Quarantine Agency (APQA) (approval no: 2018-398 and 2018-412). All procedures were carried out in a biosafety level three facility at the APQA.

### Shannon Entropy and Selective Pressure Analysis

Genomic variability between H5N8 HPAIVs derived from ducks and other hosts was evaluated by genome-wide Shannon entropy analysis using the entropy tool in the HIV sequence database (https://www.hiv.lanl.gov/content/sequence/ENTROPY/entropy.html). Shannon entropy differences (Hdiff) were calculated for each gene segment, with differences in variability considered significant when *p* < 0.05.

Gene- and site-specific selection pressures for all segments of the H5N8 viruses isolated in South Korea during 2014–2016 were measured as the ratio of non-synonymous substitutions (dN) to synonymous substitutions (dS) per site. These analyses were performed using a combination of four methods: single-likelihood ancestor counting (SLAC), fixed-effects likelihood (FEL), fast unconstrained Bayesian approximation (FUBAR), and a mixed effects model of evolution (MEME) available at the Datamonkey online version of the Hy-Phy package (19–21). The selective pressures of each gene of the C0, C1, C2, and C4 subtypes were compared and statistically significant positive selection of amino acid sites was estimated (SLAC, FEL, MEME, *p*-value < 0.1; FUBAR, posterior probability >0.9).

### Bayesian Discrete Trait Phylodynamic Analysis

Hemagglutinin (HA) gene sequences were subjected to Bayesian discrete trait phylodynamic analysis (DTA) to investigate virus transmission history among regions and host species. These analyses included all 62 available complete HA genome sequences of clade 2.3.4.4A H5N8 HPAIVs identified in wild waterfowl in East Asia during 2013–2016 that had been downloaded from the GISAID EpiFlu database (http://www.gisaid.org). An ancestral state reconstruction approach and Bayesian stochastic search variable selection (BSSVS) were utilized to determine the most probable transmission history. Geographically discrete nominal categories were assigned at the province level for viruses collected in Korea; these included “Wild Bird” (WB) (ntax = 115 sequences), “Jeollanam-do” (JN) (ntax = 115 sequences), “Jeollabuk-do” (JB) (ntax = 63 sequences), “Gyeonggi-do” (GG) (ntax = 77 sequences), ‘Gyeongsang-do' (GS) (ntax = 22 sequences), “Chungcheongnam-do” (CN) (ntax = 43 sequences), “Chungcheongbuk-do” (CB) (ntax = 69 sequences), “Gangwon-do” (GW) (ntax = 2 sequences), and “Jeju-do” (JJ) (ntax= 1 sequence). To account for potential sampling biases, sequences were subsampled to preserve diversity relative to geographic locations and host species. The WB, JN, JB, GG, and CB datasets were subsampled based on nucleotide sequence identities of 99.98, 99.9, 99.95, 99.95, and 99.95%, respectively, using CD-HIT (14). The GW and JJ datasets were excluded from the analysis because of their small sample sizes. The subsampled DTA dataset consisted of 328 sequences, including 62 WB, 57 JN, 45 JB, 55 GG, 22 GS, 43 CN, and 44 CB sequences.

DTA was also performed to determine the transmission dynamics between host species, including “Wild Bird” (ntax = 115 sequences), “Duck” (ntax = 294 sequences), “Chicken” (ntax = 71 sequences), and “minor poultry” (ntax =27 sequences). The Wild Bird, Duck, and Chicken datasets were subsampled using CD-HIT, based on nucleotide identities of 99.95, 99.87, and 100%, respectively (22). This subsampled DTA dataset consisted of 204 sequences, including 65 wild bird, 61 duck, 51 chicken, and 27 minor poultry sequences. For both analyses, Bayesian relaxed clock phylogenies were reconstructed using BEAST version 1.10.4 (23). The Hasegawa, Kishino, and Yano nucleotide substitution model with an uncorrelated log-normal distribution relaxed-clock method was used, along with a Gaussian Markov Random Field (GMRF) Bayesian skyride coalescent prior (24). The best supported viral transitions between discrete categories was identified by calculating the Bayes factor (BF) using SPREAD version 1.0.7 (25). A transition was defined as significant when BF >3 and posterior probability >0.5. The rate and number of viral transitions between discrete categories (Markov jump) and the time spent in a state between transitions (Markov reward) were estimated using stochastic mapping (26). The Markov Chain Monte Carlo (MCMC) was run in parallel for three chains, each with 100 million steps and samples across chains combined after 10% burn-in. The parameters, each of which had effective sample sizes >200, were analyzed with TRACER v1.5 (http://tree.bio.ed.ac.uk/software/tracer/). A maximum clade credibility (MCC) tree was generated using TreeAnnotator and visualized using FigTree 1.4.4 (http://tree.bio.ed.ac.uk/software/figtree/). The number of transition events and the discrete state proportion over time in monthly intervals were calculated based on analyses of posterior trees using the program PACT (http://www.trevorbedford.com/pact).

The impact of viral source/sink sample sizes on Bayesian discrete inference datasets was determined using generalized linear model (GLM) analysis as an extension of DTA (23). GLM calculates the virus migration rates as a linear combination of coefficients and coefficient indicators, and predictors. The sample size of each discrete state was used to predict the viral transition rates of two separate DTAs. The coefficient indicator and coefficient describe if and to what degree each predictor contributes to the migration rate.

## Results

### ML Phylogenetic Analysis

**Figure 1:**
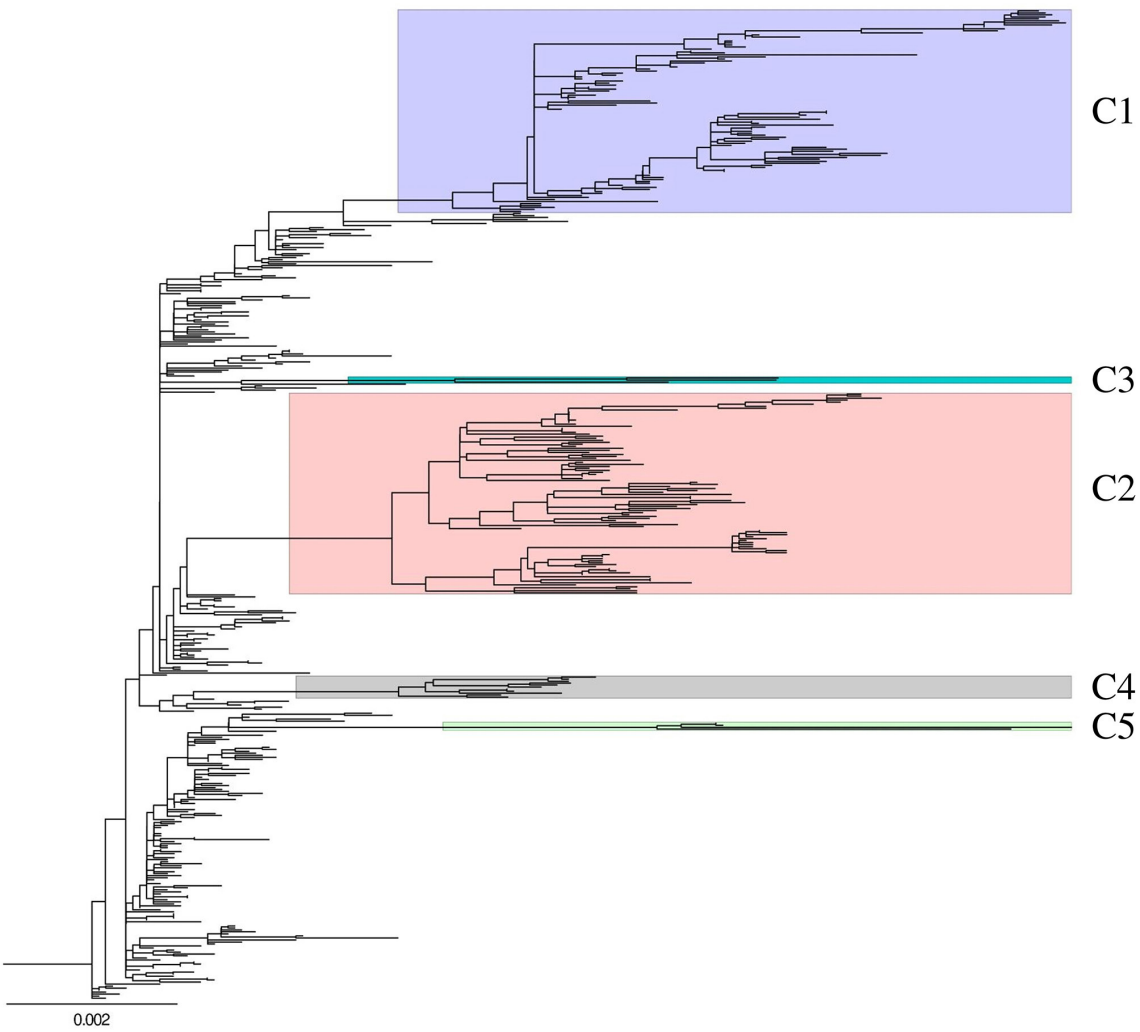
Genetic characterization of H5N8 high pathogenicity avian influenza isolated in South Korea during 2014–2016. Maximum-likelihood phylogenetic tree of concatenated complete genome sequences. Whole genome sequences of 493 H5N8 high pathogenicity avian influenza viruses collected from 388 birds on domestic poultry farms and 105 wild birds between January 2014 and April 2016. Labels and highlights indicate genetic subgroups. C3 cluster had strains isolated from wild bird in only Japan. Non-labeled and highlighted viruses are belonged to subgroup C0. Phylogenetic analyses were performed with RAxML version 8.2.12 using a gamma distribution and a general time-reversible model.

A ML phylogenetic tree of concatenated whole genome sequences of 493 H5N8 HPAIVs isolated in South Korea and Japan during 2014–2016 was generated to determine the genetic relationship among isolates (Figure 1). The viruses isolated from wild bird and poultry in South Korea were classified into multiple genetic clusters, including primitive (subgroup C0) and further classified (subgroups C1, C2, C4, and C5) viruses. Viruses of subgroup C3, which were detected in North America, Taiwan, and Japan during 2014–2015, were not detected in South Korea.

### Distribution in Geographic Region and Host

Of the 273 viruses belonging to subgroup C0 detected in South Korea from January to June 2014, 58 were detected in wild birds and 215 in domestic poultry, including in 162 ducks, 42 chickens, and 11 minor poultry (Supplementary Figure 1). These 273 viruses included viruses from JL (*n* = 82), CC (*n* = 72), GG (*n* = 41), GS (*n* = 17), GW (*n* = 2), and JJ (*n* = 1) provinces (Supplementary Figure 2). Subgroup C1 (*n* = 101) was the predominant H5N8 HPAIV isolated from poultry from September 2014 to November 2015, especially in domestic ducks (*n* = 74), with 57, 25, 12, and 3 obtained from ducks in JL, CC, GG, and GS provinces, respectively (Supplementary Figures 1, 2). Four viruses belonging to subgroup C5 were detected from May 2015 to April 2016, two in wild birds and two in domestic ducks, with all detected in GG province. Of the 87 subgroup C2 viruses detected in South Korea from December 2014 to April 2015, 18 were detected in wild birds and 69 in domestic poultry, including in 48 ducks, 19 chickens, and two minor poultry, with all detected in the western part of the country. Eight subgroup C4 viruses were detected from November 2014 to January 2015, three in wild birds and five in domestic ducks, including three in JL and two in GS province.

### Pathogenicity and Transmissibility in Chickens

**Figure 2:**
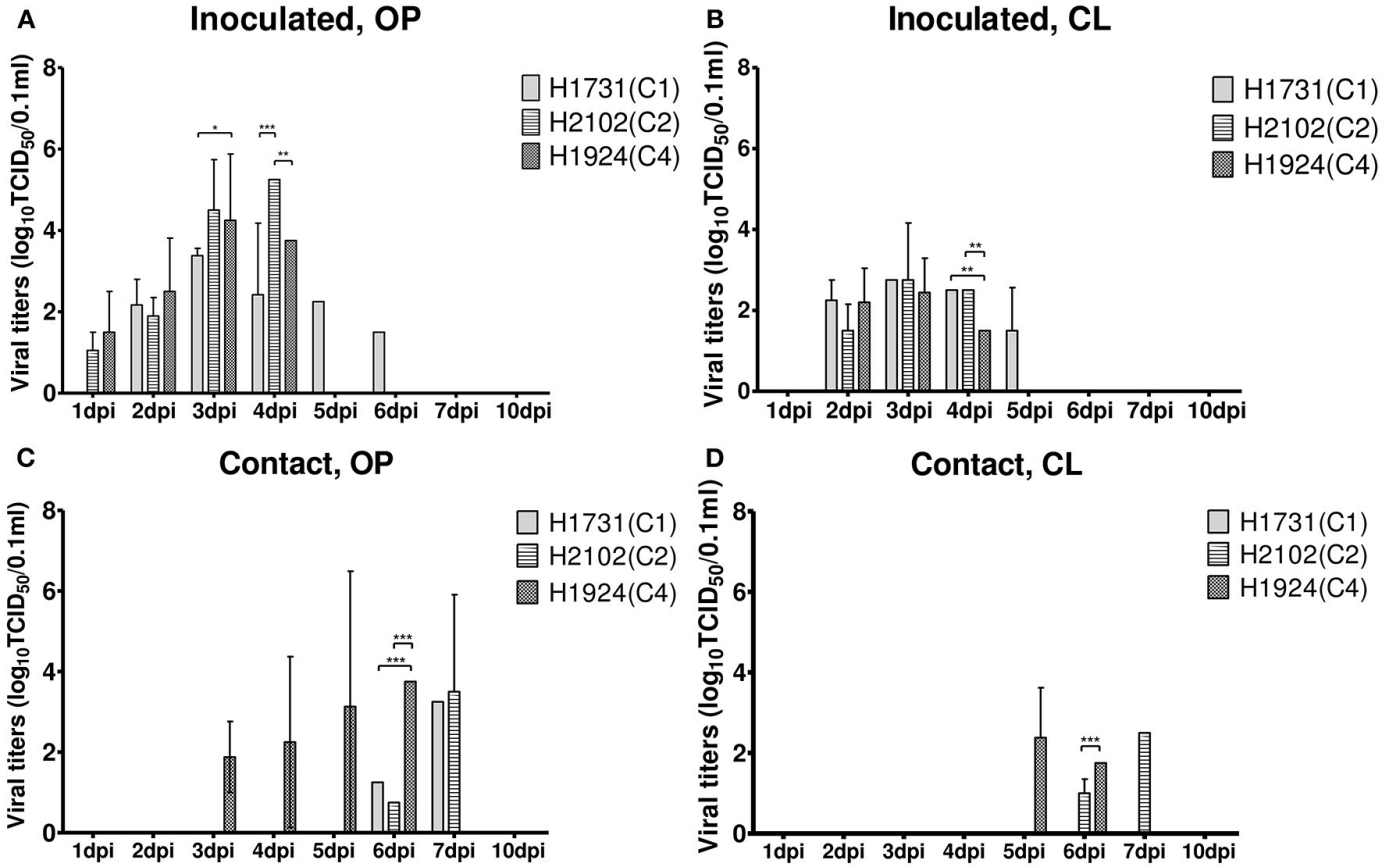
Shedding of H1731, H1924, and H2102 viruses in SPF chickens. Virus was isolated from swab samples collected from **(A,B)** chickens intranasally inoculated with 10^6^ EID_50_/0.1 ml of virus and **(C,D)** from chickens cohoused with inoculated chickens. Viral titer was measured in DF-1 cells and shown as the mean ± standard deviation. *p < 0.05, **p < 0.01, ***p < 0.001. OP, oropharyngeal; CL, cloacal; SPF, specific pathogen-free; TICD_50_, median tissue culture infectious dose.

To investigate the differences in pathobiology among viral subgroups, chickens were intranasally inoculated with H1731, H2102, or H1924 virus, defined as the index viruses of the C1, C2, and C4 subgroups, respectively (Table 1). All chickens inoculated with 10^6^ EID_50_/0.1 ml of the H1731 virus died within 6 days with a mean death time (MDT) of 5 days. By contrast, all chickens inoculated with 10^6^ EID_50_/0.1 ml of the H2102 and H1924 viruses died in less than 4 days with MDTs of 3.8 and 3.7 days, respectively. The BLD_50_ of the H1731, H2102, and H1924 viruses were 10^4.4^, 10^4.5^, and 10^3.5^ EID_50_/0.1 ml, respectively. Viral shedding of H1731 into the oropharynx and cloaca was detected from 2 to 6 dpi, whereas viral shedding of H2102 and H1924 were detected from 1 to 4 dpi (Figures 2A,B). The peak virus titers in oropharyngeal swabs were lower in H1731-inoculated chickens (3.4 log_10_ median tissue culture infectious dose [TCID_50_/0.1 ml]) than in chickens inoculated with H2102 (5.25 log_10_TCID_50_/0.1 ml) and H1924 (4.3 log_10_TCID_50_/0.1 ml). None of the surviving SPF chickens shed virus or showed an HI response.

**Table 1:** Pathogenicity and transmissibility of H1731, H2102, and H1924 in SPF chickens.

**Virus**	**Group (No. of chickens)**	**BLD_**50**_ (Log_**10**_EID_**50**_/0.1 ml)**	**MDT (days)^a^**	**Mortality (%)^b^**			
				**10^**3**^**	**10^**4**^**	**10^**5**^**	**10^**6**^**
H1731	Inoculated(5)	4.4	5.0	0/5(0)	1/5(20)	5/5(100)	5/5(100)
	Contact(3)	–	8.0	–	–	–	1/3(33)
H2102	Inoculated(5)	4.5	3.8	0/5(0)	0/5(0)	5/5(100)	5/5(100)
	Contact(3)	–	7.9	–	–	–	3/3(100)
H1924	Inoculated(5)	3.5	3.7	0/5(0)	5/5(100)	5/5(100)	5/5(100)
	Contact(3)	–	6.1	–	–	–	3/3(100)

a *MDT was measured in groups inoculated with 10^6^EID_50_/0.1 ml of H1731, H2102, and H1924 viruses*;

b *SPF chickens were intranasally inoculated with serial 10-fold dilutions, ranging from 10^3^ to 10^6^ EID_50_/0.1 ml of H1731, H2102, and H1924 viruses: BLD, Bird infectious dose; MDT, mean death time*.

Analysis of viral transmission showed that one of the three chickens cohoused with the H1731-inoculated chickens died and shed virus (1.3–3.3 log_10_TCID_50_/0.1 ml) into the oropharynx (Figures 2C,D). By contrast, all of the chickens cohoused with the H2102 and H1924-inoculated chickens died and shed virus into both the oropharynx (0.8–3.75 log_10_TCID_50_/0.1 ml) and cloaca (1.0–2.5 log_10_TCID_50_/0.1 ml). Chickens cohoused with the H1731- and H2102-inoculated chickens survived longer, with MDTs of 8 and 7.9 days, respectively, than chickens cohoused with H1924-inoculated chickens, with an MDT of 6.1 days.

**Figure 3:**
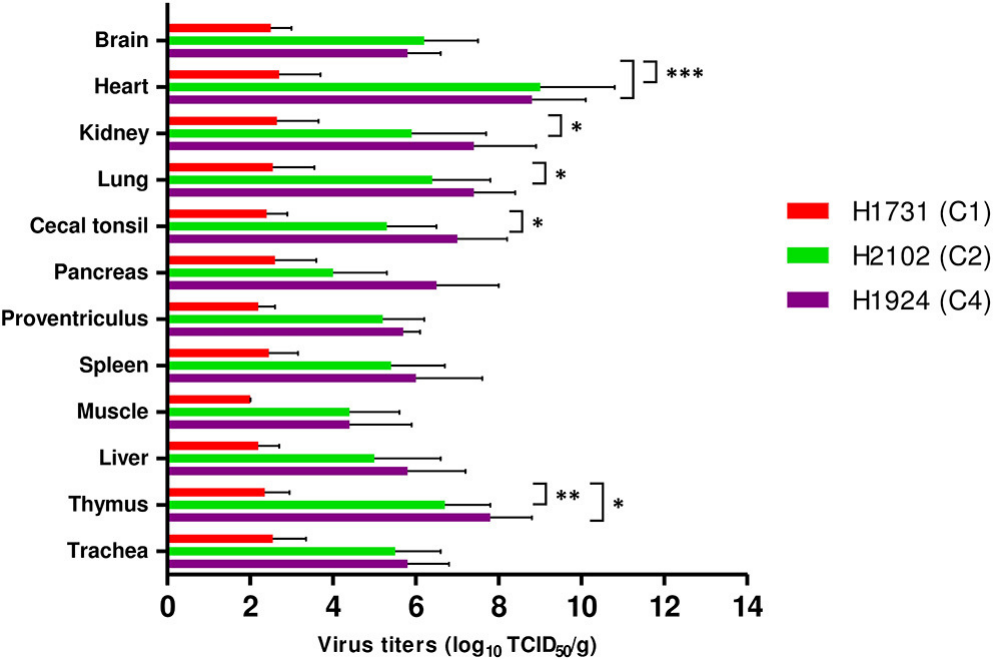
Viral titers in tissues of chickens infected with H1731, H1924, and H2102 virus. Three SPF chickens were intranasally inoculated with 10^6^ EID_50_/0.1 mL of each virus and euthanized 3 days later. Tissue homogenates were inoculated into DF-1 cells. Viral titers were expressed as median tissue culture infectious dose (TCID_50_). Data represent mean ± SD. *p < 0.05, **p < 0.01, ***p < 0.001.

All viral strains replicated in all collected tissues, including brain, heart, kidney, lung, cecal tonsil, pancreas, proventriculus, spleen, muscle, liver, thymus, and trachea (Figure 3). Viral titers in tissue samples were substantially lower in H1731-inoculated than in H2102- and H1924-inoculated chickens. In particular, the mean viral titers in the hearts of H1731-inoculated chickens were significantly lower than the viral titers in the hearts of H2102- and H1924-inoculated chickens (*p* < 0.001 each). In addition, viral titers in the kidney, lung, and cecal tonsil were significantly lower in H1731- than in H1924-inoculated chickens (*p* < 0.05 each), and viral titers in the thymus of H1731-inoculated chickens were significantly lower than titers in the thymus of chickens inoculated with H1924 (*p* < 0.01) and H2102 (*p* < 0.05) viruses.

### Shannon Entropy Analysis and Selective Pressure

Calculations of Shannon entropy for all positions of the coding regions identified 53 dominant mutations in duck-derived H5N8 HPAIVs when compared with other host-derived H5N8 viruses (Table 2). H1731 (C1) virus had multiple mutations with significance, whereas Buan2 (C0), H2102 (C2), and H1924 (C4) viruses had no related mutation. Shannon entropy analysis of H1731 (subgroup C1) virus identified 18 amino acid substitutions (T184A, A442S, and D678G in PB2; V219I in PB1; V323I, V327G, and T618K in PA; S141P, S163N, D376N, Q455K, and A528V in HA; L23I, S69N, and T329I in NA; N80S and D171E in NS1; and M14K in NS2). Entropy analysis of the Buan2 (subgroup C0), H2102 (subgroup C2), and H1924 (subgroup C4) viruses, however, did not identify any amino acid substitutions. All of the mutations were founded in subgroup C1 and took up a high proportion (92.1–100%) except for A442S of PB2 (9.9%) compared with that of the other subgroups (0–17.8%) (Supplementary Table 1).

**Table 2:** Shannon entropy analysis between H5N8 highly pathogenic avian influenza viruses isolated from duck and other hosts.

**Gene**	**nt position**	**nt change**	**nt proportion**				***p*-value**	**Entropy difference between other hosts and duck**	**Amino acid change**	**Buan2 (C0)**	**H1731 (C1)**	**H2102 (C2)**	**H1924 (C4)**
			**Other hosts**		**Duck**								
PB2	**550**	**A550G**	**A(154)^a^**	**G(37)**	**A(183)**	**G(110)**	**0**	**−0.17**	**T184A**	T	**A**	T	T
	1188	A1188T	A(189)	T(2)	A(275)	T(18)	0.01	−0.173	E396D	E	E	E	E
	**1324**	**G1324T**	**G(190)**	**T(1)**	**G(284)**	**T(9)**	**0.03**	**−0.105**	**A442S**	A	**S**	A	A
	1489	A1489C	A(169)	C(22)	A(219)	C(74)	0	−0.208	S497R	S	S	S	S
	**2033**	**A2033G**	**A(164)**	**G(27)**	**A(219)**	**G(74)**	**0**	**−0.158**	**D678G**	D	**G**	D	D
PB1	632	G632A	G(191)		G(287)	A(6)	0	−0.1	R211K	R	R	R	R
	**655**	**G655A**	**G(153)**	**A(38)**	**G(206)**	**A(87)**	**0**	**−0.109**	**V219I**	V	**I**	V	V
	1289	G1289A	G(191)		G(285)	A(8)	0.01	−0.125	R430K	R	R	R	R
	1439	A1439G	A(191)		A(286)	G(7)	0.03	−0.113	K480R	K	K	K	K
	1771	G1771A	G(191)		G(284)	A(9)	0	−0.137	V591I	V	V	V	V
	2207	A2207C	A(191)		A(287)	C(6)	0.05	−0.1	K736T	K	K	K	K
	2270	A2270G	A(190)	G(1)	A(281)	G(12)	0	−0.138	K757R	K	K	K	K
PA	176	A176T	A(191)		A(285)	T(8)	0.01	−0.125	K59I	K	K	K	K
	670	T670C	T(189)	C(2)	T(271)	C(22)	0	−0.231	S224P	S	S	S	S
	**967**	**G967A**	**G(150)**	**A(41)**	**G(198)**	**A(95)**	**0**	**−0.11**	**V323I**	V	**I**	V	V
	**980**	**T980G**	**T(190)**	**G(1)**	**T(284)**	**G(9)**	**0.03**	**−0.105**	**V327G**	V	**G**	V	V
	1010	C1010T	C(189)	T(2)	C(275)	T(18)	0.01	−0.173	A337V	A	A	A	A
	1076	A1076C	A(186)	C(5)	A(273)	C(20)	0.02	−0.128	N359T	N	N	N	N
	1159	G1159A	G(189)	A(2)	G(275)	A(18)	0.01	−0.173	V387I	V	V	V	V
	**1853**	**C1853A**	**C(165)**	**A(26)**	**C(219)**	**A(74)**	**0**	**−0.167**	**T618K**	T	**K**	T	T
	2047	C2047A	C(191)		C(288)	A(5)	0.05	−0.086	L683I	L	L	L	L
HA	**469**	**T469C**	**T(165)**	**C(26)**	**T(224)**	**C(69)**	**0**	**−0.148**	**S141P**	S	**P**	S	S
	515	C515T	C(189)	T(2)	C(280)	T(13)	0.01	−0.123	A156V	A	A	A	A
	**536**	**G536A**	**G(147)**	**A(44)**	**G(197)**	**A(96)**	**0**	**−0.093**	**S163N**	S	**N**	S	S
	610	A610G	A(189)	G(2)	A(278)	G(15)	0.01	−0.144	T188A	T	T	T	T
	633	C633A	C(191)		C(288)	A(4) T(1)	0.04	−0.095	D195E	D	D	D	D
	759	T759A	T(191)		T(284)	A(9)	0	−0.137	D236E	D	D	D	D
	865	C865A	C(191)		C(284)	A(9)	0	−0.137	H273N	H	H	H	H
	**1171**	**G1171A**	**G(151)**	**A(40)**	**G(197)**	**A(96)**	**0**	**−0.119**	**D376N**	D	**N**	D	D
	**1408**	**C1408A**	**C(164)**	**A(27)**	**C(219)**	**A(74)**	**0**	**−0.158**	**Q455K**	Q	**K**	Q	Q
	1456	T1456C	T(190)	C(1)	T(283)	C(10)	0.03	−0.116	Y471H	Y	Y	Y	Y
	1484	A1484G	A(189)	G(2)	A(278)	G(15)	0	−0.144	E480G	E	E	E	E
	1489	G1489A	G(179)	A(12)	G(250)	A(43)	0	−0.182	V482I	V	V	V	V
	**1628**	**C1628T**	**C(151)**	**T(40)**	**C(197)**	**T(96)**	**0**	**−0.119**	**A528V**	A	**V**	A	A
NP	1130	G1130A	G(191)		G(287)	A(6)	0.02	−0.1	S377N	S	S	S	S
NA	**67**	**C67A**	**C(164)**	**A(27)**	**C(219)**	**A(74)**	**0**	**−0.158**	**L23I**	L	**I**	L	L
	**206**	**G206A**	**G(162)**	**A(29)**	**G(219)**	**A(74)**	**0**	**−0.139**	**S69N**	S	**N**	S	S
	220	G220A	G(190)	A(1)	G(285)	A(8)	0.05	−0.093	V74I	V	V	V	V
	239	G239A	G(189)	A(2)	G(275)	A(18)	0.01	−0.173	G80D	G	G	G	G
	**986**	**C986T**	**C(162)**	**T(29)**	**C(210)**	**T(83)**	**0**	**−0.17**	**T329I**	T	**I**	A	T
	1174	G1174A	G(191)		G(283)	A(10)	0	−0.149	V392M	V	V	V	V
NS1	167	C167A	C(189)	A(2)	C(274)	A(18) T(1)	0.01	−0.195	T56I	T	T	T	T
	175	C175T	C(182)	T(9)	C(260)	T(33)	0.02	−0.162	R59C	R	R	R	R
	226	G226A	G(189)	A(2)	G(276)	A(17)	0.01	−0.163	A76T	A	A	A	A
	**239**	**A239G**	**A(162)**	**G(29)**	**A(211)**	**G(82)**	**0**	**−0.167**	**N80S**	N	**S**	N	N
	409	A409G	A(191)		A(284)	G(9)	0.01	−0.137	I137V	I	I	I	I
	**513**	**T513A**	**T(166)**	**A(25)**	**T(221)**	**A(68) C(3) G(1)**	**0**	**−0.23**	**D171E**	D	**E**	D	D
	614	G614T	G(191)		G(286)	A(1) T(6)	0.03	−0.123	S205I	S	S	S	S
	625	G625A	G(189)	A(2)	G(275)	A(18)	0.01	−0.173	D209N	D	D	D	D
	637	T637C	T(188)	C(2) Y(1)	T(275)	C(18)	0.03	−0.14	S213P	S	S	S	S
NS2	**41**	**T41A**	**T(166)**	**A(25)**	**T(221)**	**A(68) C(3) G(1)**	**0**	**−0.23**	**M14K**	M	**K**	M	M
	142	G142T	G(191)		G(286)	A(1) T(6)	0.03	−0.123	A48S	A	A	A	A
	242	A242G	A(191)		A(284)	G(9)	0	−0.137	E81G	E	E	E	E

a *Nucleotide sequence at a significant nucleotide position (the number of H5N8 HPAI viruses having that mutation). Bold indicate the significant mutation that exclusively detected in H1731 virus*.

Analyses of the selection profiles of the H5N8 HPAIVs identified individual codons under positive selection pressure (Supplementary Table 2). Nine amino acids (aas) in the C0 subgroup, including aa 694 of PB2, aas 219 and 757 of PB1, aas 181 and 269 of HA [H5 numbering], aas 11 and 13 of M2, and aas 109 and 180 of NS1, were identified as being under positive selection. Eight aas in the C1 subgroup, including aa 184 and 559 of PB2, aas 219 and 757 of PB1, aa 113 of NA, aas 13 and 19 of M2, and aa 59 of NS1; five aas in the C2 subgroup, including aas 219 and 757 of PB1, aas 183 and 353 of NP, and aa 369 of NA; and two aas in the C4 subgroup, including aas 105 and 331 of NP, were also identified as being under positive selection pressure.

### Bayesian Phylogenetic Analysis of the HA Gene

**Figure 4:**
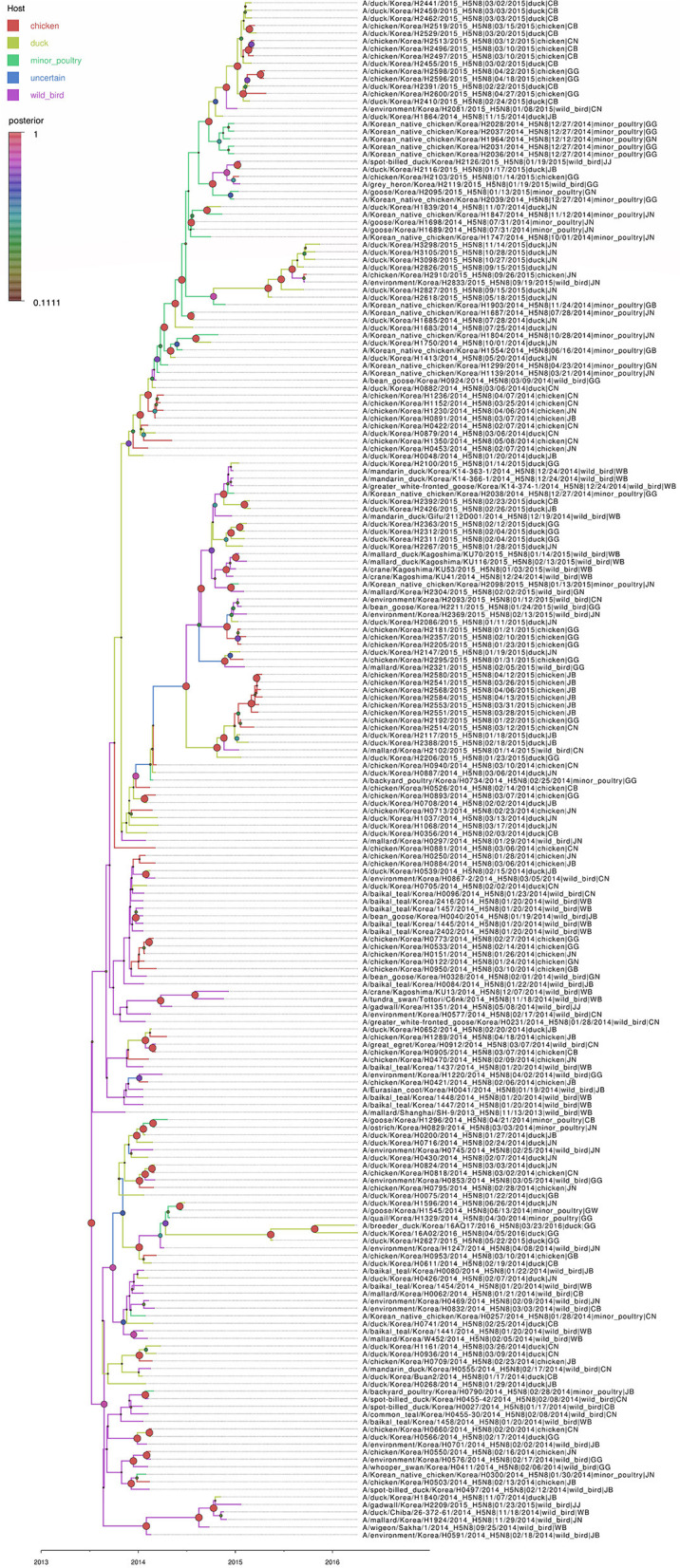
Bayesian phylogenetic tree of the hemagglutinin gene of H5N8 high pathogenicity avian influenza viruses isolated in South Korea during 2014–2016. The host species were defined as discrete states. Posterior probabilities of nodes are indicated by the sizes and colors of the circles.

Based on the Bayesian phylogeny of HA gene, the time to most recent common ancestor (tMRCA) of all viruses identified in Korea was estimated to have arisen on July 2, 2013 (95% HPD: May 2 to August 11, 2013, with a posterior probability of 1), indicating that the H5N8 viruses of wild bird origin in South Korea most likely emerged during the breeding season of wild waterfowl in 2013 (Figure 4). The mean substitution rate of the HA gene was calculated as 7.12 x 10^−3^ substitutions/site/year (95% BCI: 5.53 x 10^−3^ to 8.80 x 10^−3^), within the global range for AIV substitution rate (27).

### Host Dynamics

**Figure 5:**
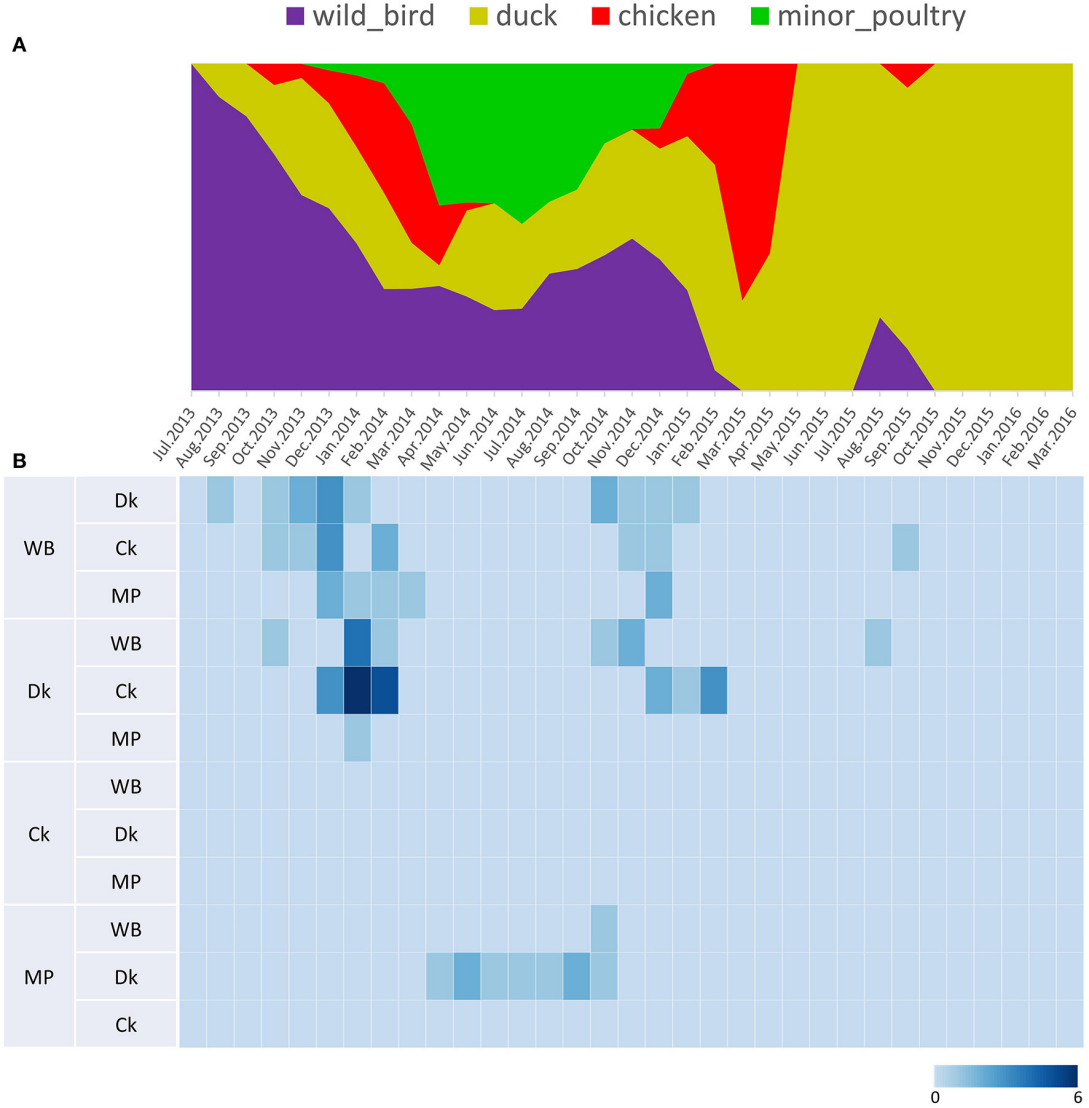
Transition events and discrete state proportion over time of H5N8 high pathogenicity avian influenza viruses isolated in South Korea during 2014–2016. **(A)** Proportion of ancestral discrete states estimated on the phylogenetic trunk of H5N8 viruses over time. The host species were defined as discrete states. Shaded areas represent estimated ancestral discrete state proportions of each state. At each point in time, the width (y-axis) represents the mean proportion from 0 to 100% of each state. **(B)** Heat map showing the number of transition events between each discrete state (0–6) over time.

**Figure 6:**
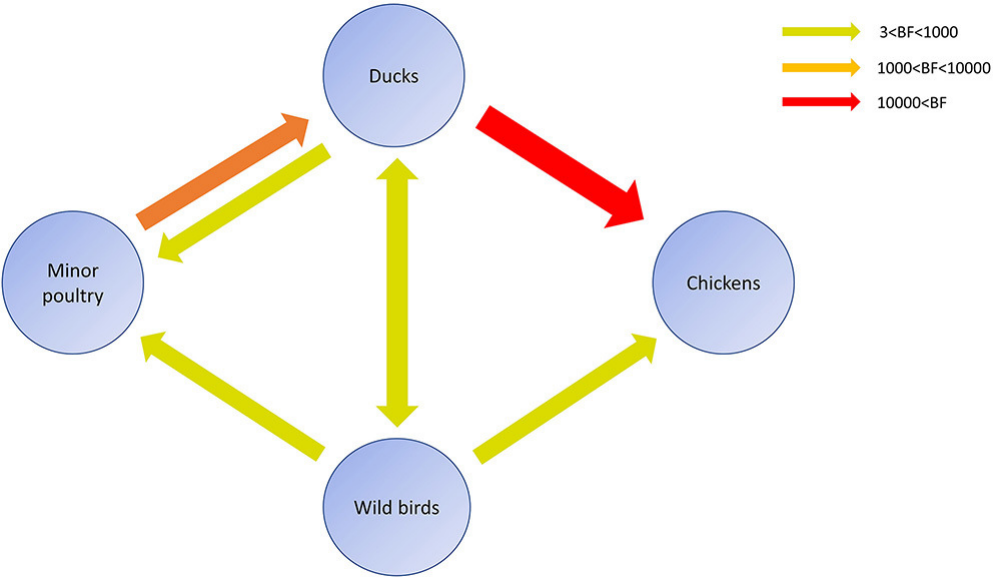
Inferred transmission networks of H5N8 viruses between host species. Arrows show the directions of viral transmission. Line colors indicate the overall Bayes Factor test support for epidemiological linkage. Red, orange, and green lines indicate statistical support with BF >10,000 (very strong support), 1,000< BF <10,000 (strong support), and 3< BF <1,000, respectively.

**Figure 7:**
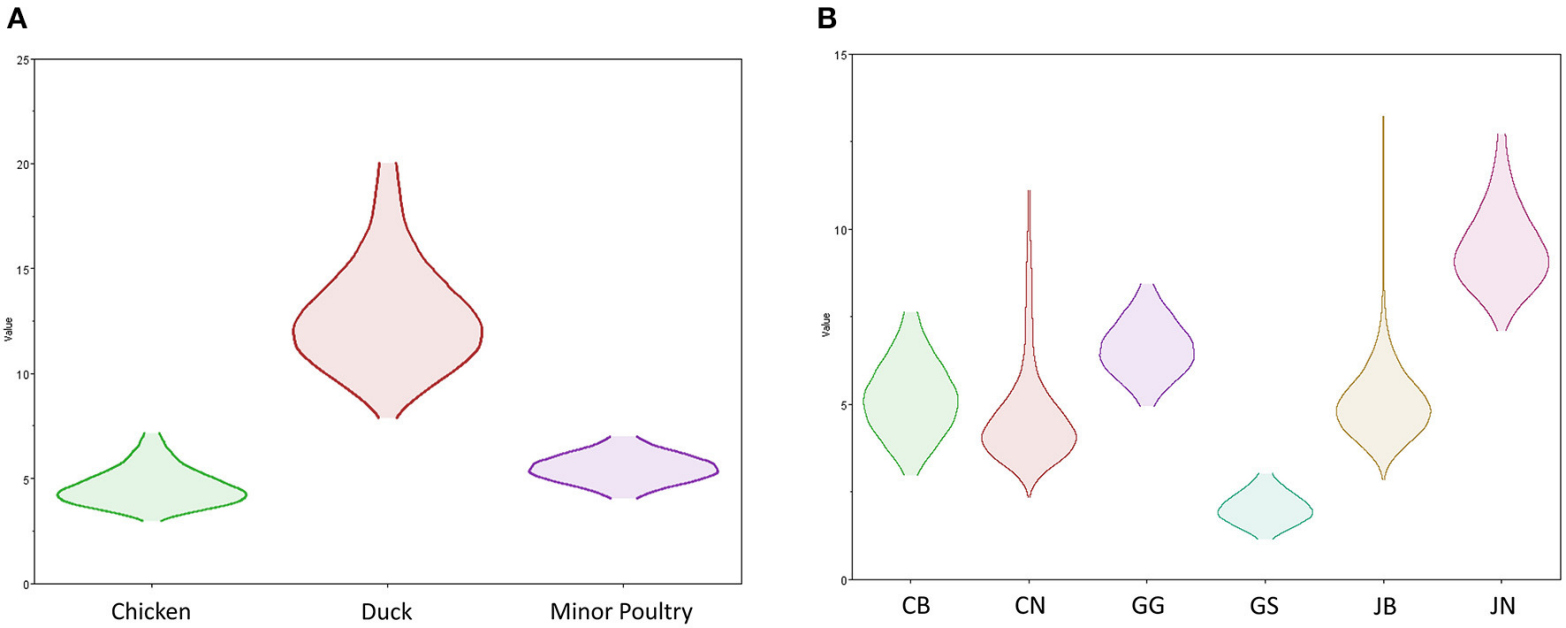
Transmission dynamics between host species and geographical region of H5N8 high pathogenicity avian influenza viruses isolated in South Korea during 2014–2016 (Markov rewards). **(A)** The host species and **(B)** regions were defined as discrete states. Markov rewards were calculated for each posterior tree using a non-reversible continuous-time Markov chain model.

Source sink dynamics of the H5N8 HPAIVs identified in South Korea during 2014–2016 were analyzed by estimating the transition rate (TR), Markov jump count, and Markov rewards. Based on our DTA, well-supported transitions from wild birds to domestic ducks (TR: 1.525, BF: 84.5189, posterior probability: 0.9741), chickens (TR: 0.625, BF: 21.7051, posterior probability: 0.9061), and minor poultry (TR: 0.615, BF: 229.3770, posterior probability: 0.9903), indicate that multiple virus introductions from wild birds to poultry species occurred mainly from August 2013 to March 2014 and from October 2014 to January 2015 (Figures 5, 6; Table 3). The well-supported transitions from domestic ducks to wild birds (TR: 1.065, BF: 79.3385, posterior probability: 0.9724), chickens (TR: 1.772, BF: 27329.6487, posterior probability: 0.9999), and minor poultry (TR: 0.251, BF: 3.5673, posterior probability: 0.6133) suggest the subsequent spread of viruses from domestic ducks to other species, including reverse spread to wild birds, during the winters of 2013–2014 and 2014–2015. Interestingly, minor poultry also contributed to virus spread, transmitting virus to domestic ducks (TR: 1.473, BF: 2275.4089, posterior probability: 0.9990). The estimated total Markov reward time for each discrete state of poultry was estimated to be highest in domestic ducks (13.093, 95% HPD: 7.9203–20.0282), followed by minor poultry (5.499, 95% HPD: 4.0152–7.0553), and chickens (4.757, 95% HPD: 2.9123–7.0446) (Figure 7A). Collectively, these results indicate that domestic ducks and minor poultry played major roles in the maintenance and transmission of H5N8 HPAIV in South Korea after its multiple introductions by wild birds. GLM analysis revealed that sample size had a negligible effect on potential bias toward the origin or destination state in this analysis (source indicator: 0.156, source coefficient: 0.05453, sink indicator: 0.43, sink coefficient: 0.185).

**Table 3:** Viral transition of discrete traits (based on HA gene sequences) between different host species (ducks, wild birds, chickens, minor poultry) in South Korea.

**Transition from**	**Transition to**	**Mean actual migration rate^a^ (95% HPD)**	**Bayes factor**	**Posterior probability**
Wild birds	Ducks	1.525 (0–3.0507)	84.5187	0.9741
Wild birds	Chickens	0.625 (0–1.5413)	21.7051	0.9061
Wild birds	Minor poultry	0.615 (0–1.2804)	229.3770	0.9903
Ducks	Wild birds	1.065 (0–2.3196)	79.3385	0.9724
Ducks	Chickens	1.772 (0.4169–3.421)	27329.6487	0.9999
Ducks	Minor poultry	0.251 (0–0.8906)	3.5673	0.6133
Minor poultry	Ducks	1.473 (0.3051–2.8596)	2275.4089	0.9990

a *Actual migration rates were calculated as the rate x indicator. HPD, highest probability density*.

### Transmission History Between Regions

**Figure 8:**
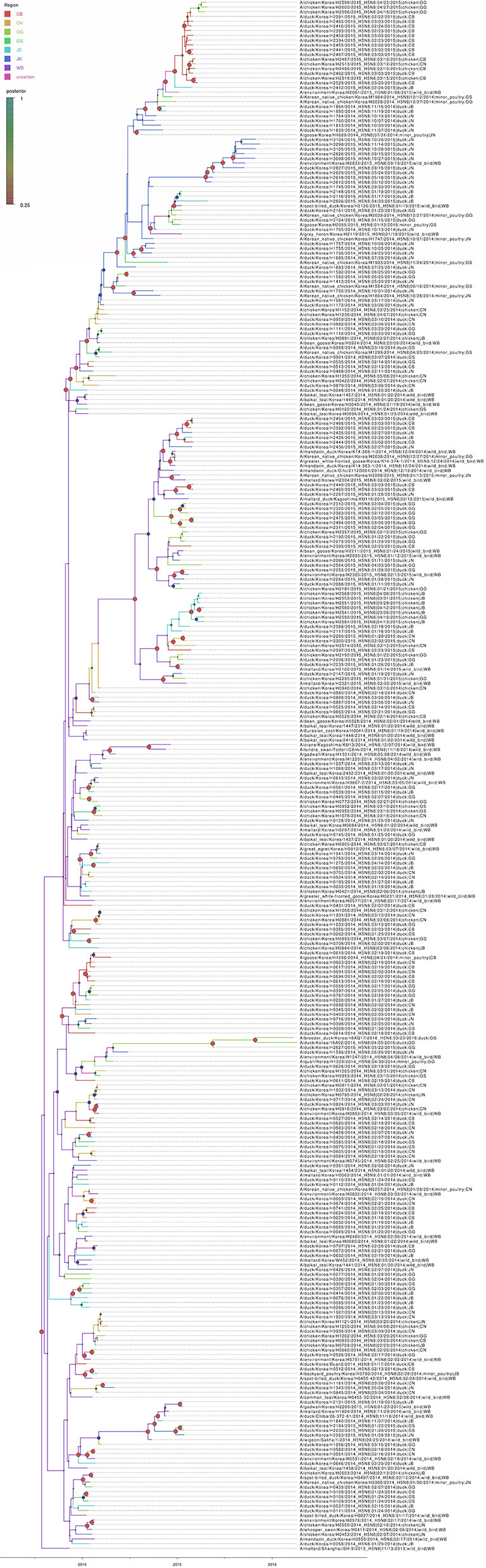
Bayesian phylogenetic tree of the hemagglutinin gene of H5N8 high pathogenicity avian influenza viruses isolated in South Korea during 2014–2016. The geographic regions were defined in the analysis as discrete states. Posterior probabilities of nodes are indicated by the sizes and colors of the circles.

**Figure 9:**
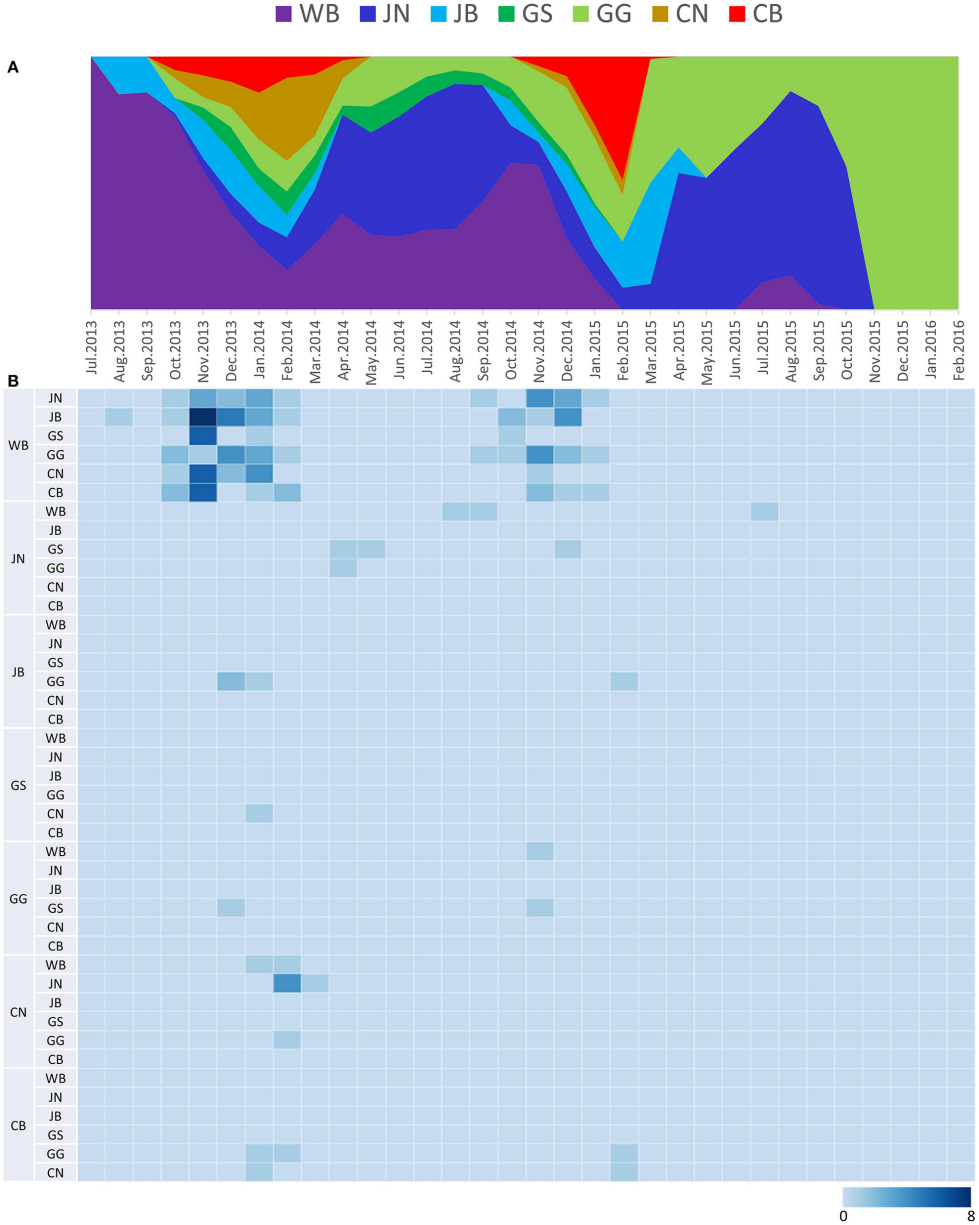
Transition events and discrete state proportion over time of H5N8 high pathogenicity avian influenza viruses isolated in South Korea during 2014–2016. **(A)** Proportion of ancestral discrete states estimated on the phylogenetic trunk of H5N8 viruses over time. Geographic regions were defined as discrete states. Shaded areas represent estimated ancestral discrete state proportions of each state. At each point in time, the width (y-axis) represents the mean proportion from 0 to 100% of each state. **(B)** Heat map showing the number of transition events between each discrete state over time (0–8).

**Figure 10:**
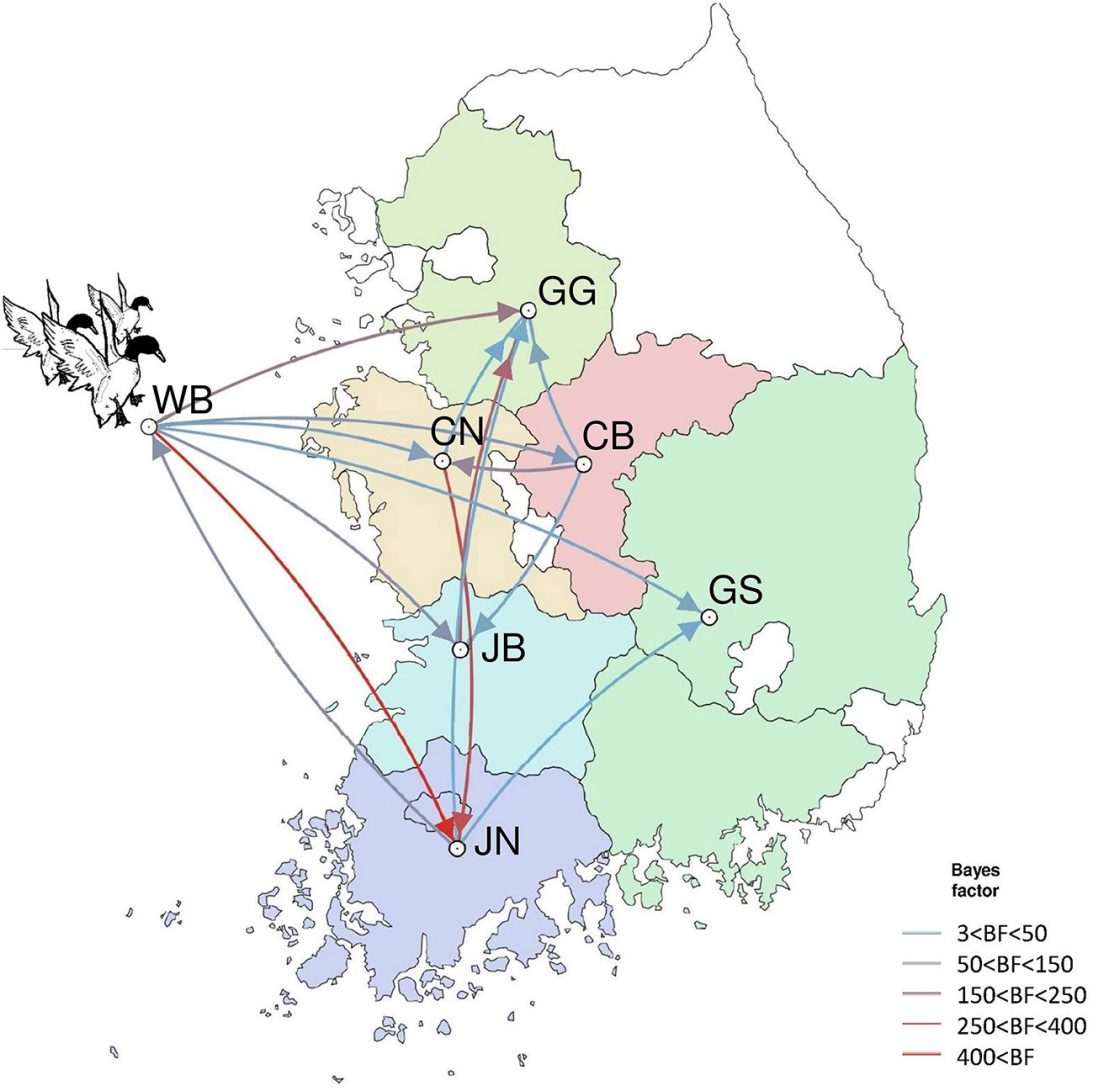
Inferred transmission networks of H5N8 viruses between regions. Arrows show the directions of transmission. Line colors indicate the overall Bayes Factor test support for epidemiological linkage. WB, wild bird; JB, Jeollabuk-do; JN, Jeollanam-do; GG, Gyeonggi-do; GS, Gyeongsang-do; CN, Chungcheongnam-do; CB, Chungcheongbuk-do.

The DTA among provinces in South Korea involved seven discrete nominal categories: CB, CN, GG, GS, JB, JN, and WB. GLM analysis showed that sample size had a negligible effect on potential bias toward the origin or destination state in this analysis (source indicator: 0.13, source coefficient: 0.0601, sink indicator: 0.388, sink coefficient: 0.0996). The DTA indicated multiple introductions of H5N8 HPAIV from wild birds into domestic poultry in various regions in South Korea (Figures 8–10; Table 4). DTA also showed that virus was transmitted from WB to JB (TR: 1.836, BF: 115.3142, posterior probability: 0.9563) in August 2013, followed by introductions into JB and JN (TR: 1.628, BF: 549.5709, posterior probability: 0.9905), GS (TR: 0.724, BF: 25.2402, posterior probability: 0.8271), GG (TR: 1.559, BF: 229.3154, posterior probability: 0.9775), CN (TR: 1.089, BF: 44.8964, posterior probability: 0.8949), and CB (TR: 1.258, BF: 88.2501, posterior probability: 0.9436) during the winters of 2013–2014 and 2014–2015, indicating multiple introductions of virus from WB into poultry farms in various regions throughout the country. Viral transmission from JN to GG (TR: 0.359, BF: 5.4350, posterior probability: 0.5075), GS (TR: 0.497, BF: 13.4123, posterior probability: 0.7177), and WB (TR: 1.013, BF: 127.8281, posterior probability: 0.9604); from JB to GG (TR: 0.97, BF: 294.0055, posterior probability: 0.9824); from CN to GG (TR: 0.709, BF: 14.1282, posterior probability: 0.7281) and JN (TR: 1.219, BF: 396.1989, posterior probability: 0.9869); and from CB to JB (TR: 0.674, BF: 24.9877, posterior probability: 0.8257), GG (TR: 742, BF: 32.0996, posterior probability: 0.8589), and CN (TR: 0.943, BF: 160.1564, posterior probability: 0.9681) suggest subsequent spread of the viruses from JN, JB, CN, and CB to the other regions during the spring of 2014 and the winters of 2013–2014 and 2014–2015, including reverse spread from JN to wild birds during the summers of 2014 and 2015. The total Markov reward time for each discrete province was the highest for JN (9.616, 95% HPD: 7.0954–12.7019), followed by GG (6.638, 95% HPD: 4.9447–8.4321), JB (5.713, 95% HPD: 2.8411–13.2159), CB (5.263, 95% HPD: 2.9685–7.6259), CN (5.223, 95% HPD: 2.3518–11.09), and GS (2.048, 95% HPD: 1.1438–3.0173) (Figure 7B). The Markov reward time and viral transmission patterns indicate that poultry in JN, JB, CN, and CB may have acted as seeding populations of H5N8 HPAIV within South Korea.

**Table 4:** Viral transition of discrete traits (based on HA gene sequences) between different regions (WB, JN, JB, GG, GS, CB, and CN) in South Korea.

**Transition from**	**Transtion to**	**Mean actual migration rate^a^ (95% HPD)**	**Bayes factor**	**Posterior probability**
WB	GS	0.724 (0–1.5599)	25.2402	0.8271
WB	CN	1.089 (0–2.1055)	44.8964	0.8949
WB	CB	1.258 (0–2.2904)	88.2501	0.9436
WB	JB	1.836 (0–3.1339)	115.3142	0.9563
WB	GG	1.559 (0–2.7841)	229.3154	0.9775
WB	JN	1.628 (0.4315–3.0045)	549.5709	0.9905
JN	GG	0.359 (0–1.3737)	5.4350	0.5075
JN	GS	0.497 (0–1.4033)	13.4123	0.7177
JN	WB	1.013 (0–2.1474)	127.8281	0.9604
JB	GG	0.97 (0–2.0953)	294.0055	0.9824
CN	GG	0.709 (0–1.9503)	14.1282	0.7281
CN	JN	1.219 (0–2.5162)	396.1989	0.9869
CB	JB	0.674 (0–1.7051)	24.9877	0.8257
CB	GG	0.742 (0–1.8076)	32.0996	0.8589
CB	CN	0.943 (0–2.1502)	160.1564	0.9681

a *Actual migration rates were calculated as the rate x indicator. HPD, highest probability density*.

## Discussion

AIVs evolve through the gradual accumulation of mutations and genome reassortment. H5N8 HPAIVs likely had opportunities to reassort with various AIV gene pools during the 28 months of the H5N8 HPAI outbreak in 2014–2016 in South Korea, which could result in the generation of novel genotypes. However, there was no evidence of reassortment with other AIVs. ML phylogenetic analysis showed that the C0 subgroup had diverged into multiple subgroups within South Korea. The detection of four distinct subgroups, C1, C2, C4, and C5, diverging from C0 viruses in the phylogenetic tree suggests that each subgroup had evolved independently within South Korea during 2014–2016 without any reassortment. These findings indicate that the H5N8 HPAIV genotype introduced into South Korea was a fixed genome constellation with a high fitness produced in the Eurasian AIV gene pool. Interestingly, the H5N8 HPAIV spread into North America in late 2014 and reassorted with North American low-pathogenicity AIVs to produce the novel Eurasian-North American reassortant H5N1, H5N2, and H5N8 viruses (28).

The representative challenge viruses of the C1, C2, and C4 subgroups differed significantly in infectivity, transmissibility, and pathogenicity in SPF chickens. H1731 (C1) had a longer MDT, significantly lower viral titers in tissues, and lower transmissibility in chickens compared with H2102 (C2) and H1924 (C4). The virulence of H1731 in chickens was also lower than that of the buan2 (the index virus of C0) (29). In particular, the amounts of H1731 virus shed into the oropharynx and cloaca of inoculated SPF chickens ranged from 10^1.5^ to 10^3.4^ (TCID_50_/0.1 ml) and from 10^1.5^ to 10^2.8^ (TCID_50_/0.1 ml), respectively, whereas the amounts of Buan2 virus shed into these tissues ranged from 10^4.2^ to 10^6.3^ (TCID_50_/0.1 ml) and from 10^3.1^ to 10^7.5^ (TCID_50_/0.1 ml), respectively. Viral titers in tissue samples were also lower in chickens inoculated with H1731 than with buan2 virus (30). These findings suggest that viruses of the C1 subgroup have lower adaptation and transmissibility in chickens. Because viruses of the C1 subgroup had circulated mainly in domestic duck farms, they likely adapted to domestic ducks. AIVs acquire diverse mutations during adaptation to a particular host (31, 32). Shannon entropy analysis identified several dominant amino acid substitutions in H5N8 HPAIVs from domestic ducks compared with viruses from other species, with H1731 being the only challenge virus to have amino acid substitutions, further indicating that viruses of the C1 subgroup had become adapted to domestic ducks.

Long-term circulation of AIVs in a certain host species allows viruses to adapt to that species, affecting their virulence and host specificity via genetic evolution. Two amino acid residues (positions 219 and 757 in PB1) in C0, C1, and C2 were found to be under positive selection pressure, with Shannon entropy analysis showing significant mutations at these positions in H5N8 HPAIVs from ducks. Because viruses of the C0, C1, and C2 subgroups mainly circulated in domestic duck farms during the 2014–2015 outbreak, these results suggest that these viruses may have undergone positive selection in domestic ducks. Amino acid substitutions that occurred during the adaptation of H5 HPAIVs to a particular host were found to be related to viral pathogenicity in that host (33). H5N8 HPAIVs have been reported to be transmitted efficiently, with subclinical infection in domestic ducks contributing to the silent spread of virus to other hosts (34, 35). The linkage between long-term circulation of H5N8 HPAIV and asymptomatic infection in domestic ducks during the 2014–2016 outbreak has not been fully determined. Analyses of the putative role of selected amino acid substitutions in domestic ducks would require a determination of the molecular mechanism underlying viral adaptation to host and changes in pathobiology.

The estimated mean tMRCA of all H5N8 HPAIVs identified in Korea was July 2013, indicating that the H5N8 HPAIVs of wild bird origin detected in South Korea most likely emerged during the breeding season of wild waterfowl in 2013. These findings were consistent with other studies on the estimated tMRCA of H5N8 HPAIV (36, 37). Phylogeographic analysis suggests multiple introductions of the H5N8 HPAIVs from wild birds into JB province, followed by subsequent spread to other provinces in South Korea during the winters of 2013–2014 and 2014–2015. These findings highlight the major role of JN, JB, CN, and CB provinces in dissemination and persistence of the virus after its introductions from wild birds. The regional proportion of ancestral discrete states through time and Markov reward analyses also indicate that the H5N8 viruses were maintained predominantly in western South Korea, specifically in JN, JB, GG, CN, and CB provinces (Supplementary Figure 3A).

The western region of South Korea contains abundant habitats for wild waterfowl and a high density of domestic duck farms (8), creating an environment vulnerable to the transmission of HPAIVs (38, 39). In addition, surveillance studies have suggested that domestic ducks are a major contributor to the spread and maintenance of HPAIVs in South Korea (40). Consistent with previous findings, the present study showed that the transmission of H5N8 HPAIV from domestic ducks to other host species occurred frequently, further indicating that domestic ducks play major roles in the maintenance, amplification, and spread of HPAIVs (38–40). Although a previous study reported unidirectional transmission of H5N8 viruses from wild waterfowl to domestic ducks (40), the present study found exchange of the viruses between domestic ducks and wild birds (Supplementary Figure 3B). Geographical transmission dynamics showed dissemination of the virus into wild birds from JN, an area with large numbers of wild bird habitats adjacent to domestic duck farms. These findings suggest the need for enhanced levels of surveillance and biosecurity measures at domestic duck farms in this region to effectively monitor and prevent the introduction and spread of HPAIV.

Viral phylodynamic analysis also showed exchanges of HPAIVs between minor poultry populations and domestic ducks in South Korea, indicating that minor poultry may serve as reservoirs to maintain and disseminate HPAIV. Minor poultry are generally raised in small outdoor operations and backyards, and are usually marketed in live bird markets (LBMs), highlighting their importance as intermediary hosts in virus transmission under poor biosecurity conditions (41). LBMs include a wide variety of live poultry species, providing an ideal environment for the introduction, maintenance, and adaptation of viruses, as well as potential conditions for transmissions between duck farms and chicken farms (40).

Collectively, the present study presents unique molecular epidemiology and pathobiology of the H5N8 HPAIV outbreak in 2014–2016 in South Korea. Our data showed multiple introductions of the H5N8 HPAIV isolated in South Korea during 2014-2016 from wild waterfowl to poultry farms in multiple provinces. The virus, initially introduced into the western part of South Korea, which contains large populations of domestic ducks, was subsequently disseminated into other regions throughout the country. Furthermore, domestic ducks likely played a pivotal role in the persistent circulation of the virus, with minor poultry also serving as a source population. In addition, sequence analysis and *in vivo* experiments support that the possible adaptation of H5N8 HPAIV in domestic ducks likely reduced its virulence in chickens. Enhanced genomic surveillance and pathobiological characterization of the viruses are essential for better understanding of HPAI epidemiology and the design of prevention strategies.

## Data Availability Statement

The datasets presented in this study can be found in online repositories. The names of the repository/repositories and accession number(s) can be found at: GISAID EpiFlu database (http://www.gisaid.org) and accession no. EPI_ISL_157609-EPI_ISL_410213.

## Ethics Statement

Experiments in animals were reviewed and approved by the Institutional Animal Care and Use Committee of the Animal and Plant Quarantine Agency (APQA) (approval no: 2018-398 and 2018-412).

## Author Contributions

Y-GB: writing. Y-JL: supervision and funding acquisition. E-KL: project administration. D-HL: writing—review and editing. Y-JS and Y-RP: NGS sequencing. DC: host transition analysis. J-HK: posterior tree analysis. G-BH: virus isolation. All authors have read and agreed to the published version of the manuscript.

## Funding

This work was supported by the Animal and Plant Quarantine Agency (APQA) and Ministry of Agriculture, Food and Rural Affairs of South Korea (MAFRA), via grant B-1543418-2021-23-01.

## Conflict of Interest

The authors declare that the research was conducted in the absence of any commercial or financial relationships that could be construed as a potential conflict of interest.

## Publisher's Note

All claims expressed in this article are solely those of the authors and do not necessarily represent those of their affiliated organizations, or those of the publisher, the editors and the reviewers. Any product that may be evaluated in this article, or claim that may be made by its manufacturer, is not guaranteed or endorsed by the publisher.

## Supplementary Material

Supplementary Figure 1 Distribution of the H5N8 HPAIV isolated in South Korea during 2014-2016.

Supplementary Figure 2 Geographical distribution of the H5N8 HPAIV isolated in South Korea during 2014-2016.

Supplementary Figure 3 Transmission dynamics between discrete states. **(A)** Inferred mean transmission matrix of H5N8 viruses between the provinces. **(B)** Inferred mean transmission matrix of H5N8 viruses between the hosts.

Supplementary Table 1 Amino acid differences between subgroups and proportion of H1731 mutation having a significance in Shannon entropy.

Supplementary Table 2 Selective pressure analysis of H5N8 highly pathogenic avian influenza viruses isolated in South Korea during 2014-2016.
