# Supplementary figures and images for: Evolution, Transmission, and Pathogenicity of High Pathogenicity Avian Influenza Virus A (H5N8) Clade 2.3.4.4, South Korea, 2014–2016
Source: Front Vet Sci. 2022 Jun 21;9:906944. doi: 10.3389/fvets.2022.906944 (PMC9253604; doi:10.3389/fvets.2022.906944)

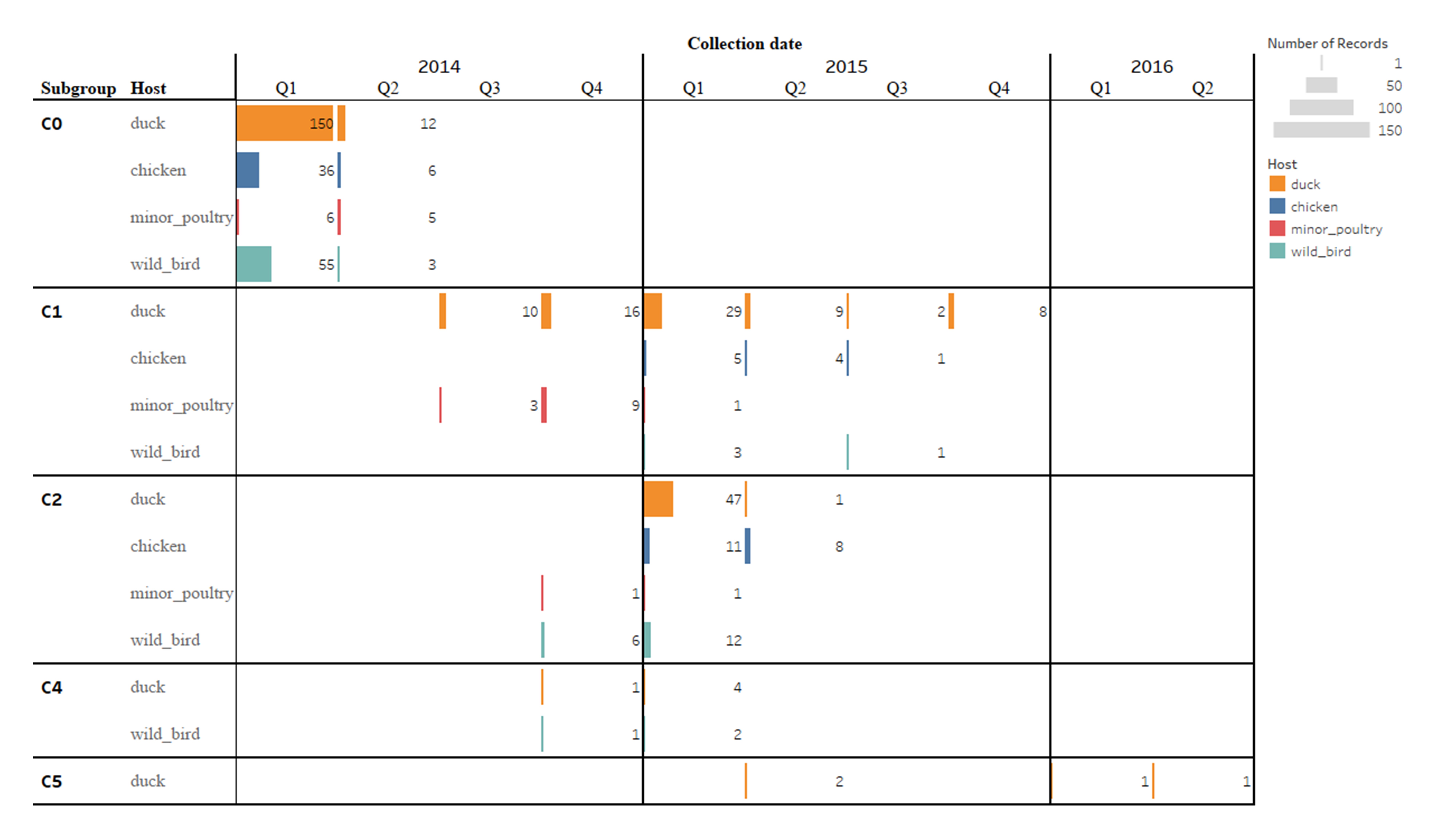

Supplement: Supplementary Figure 1 — Distribution of the H5N8 HPAIV isolated in South Korea during 2014-2016. [file Image_1.JPEG]

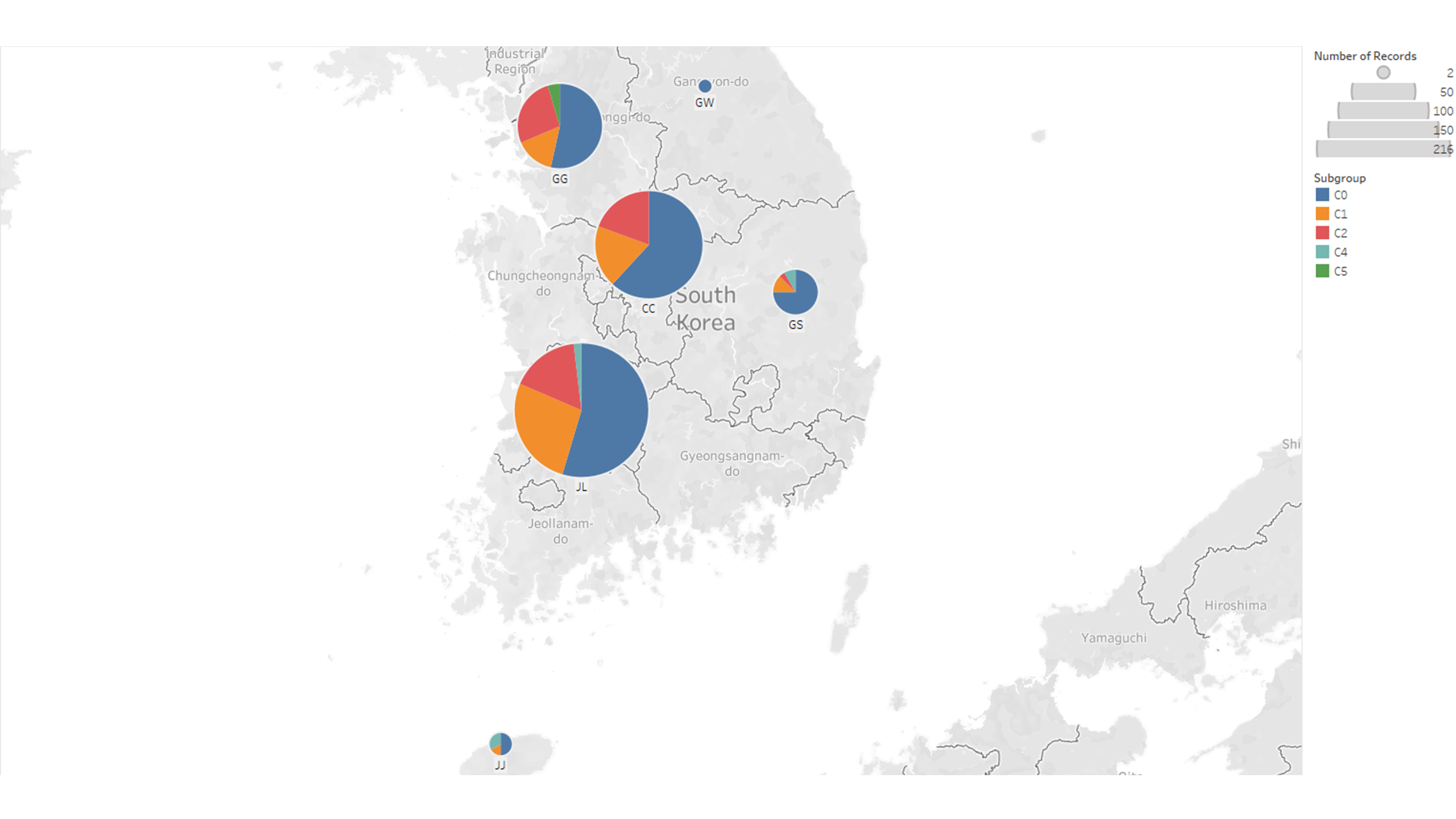

Supplement: Supplementary Figure 2 — Geographical distribution of the H5N8 HPAIV isolated in South Korea during 2014-2016. [file Image_2.JPEG]

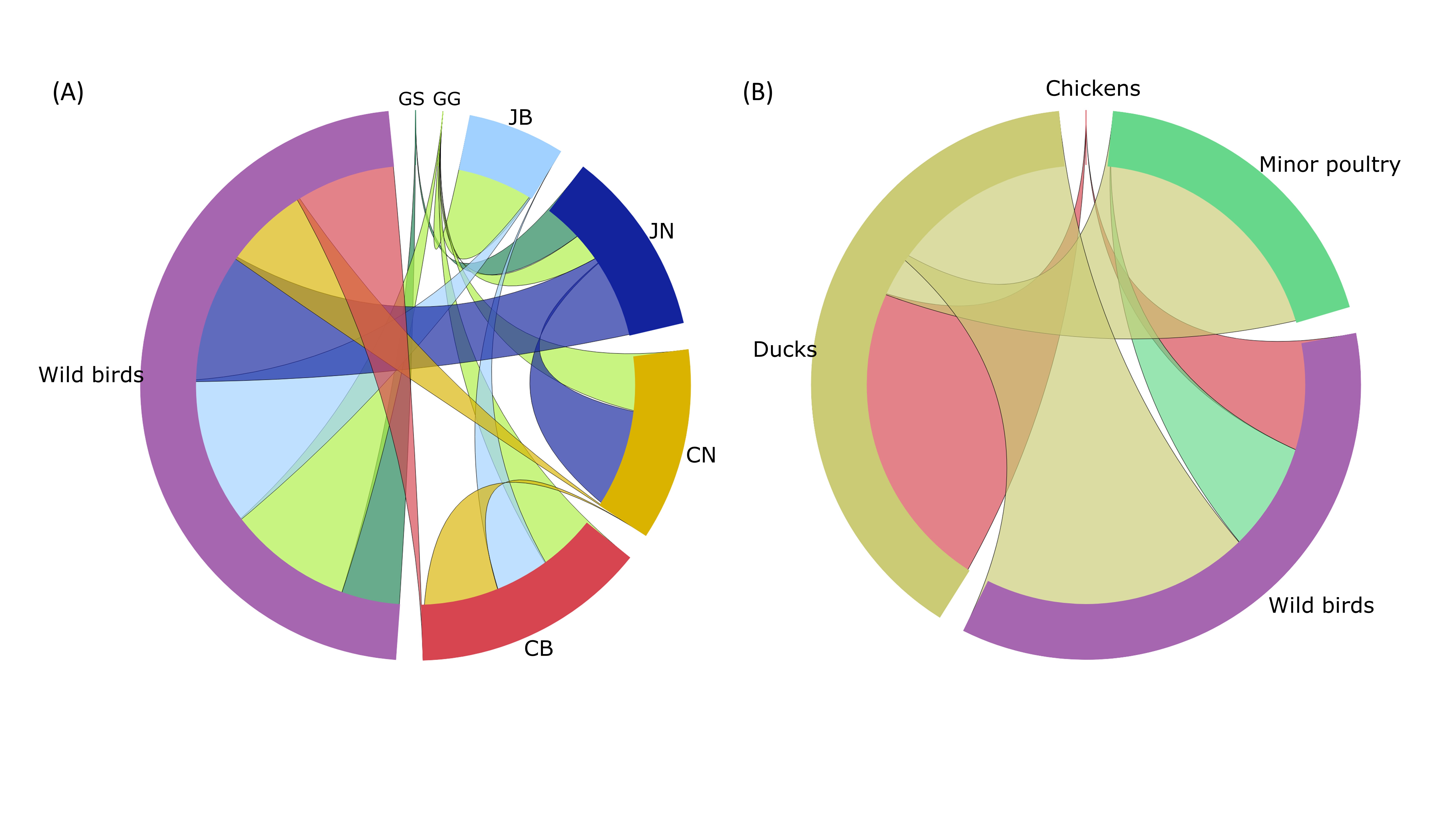

Supplement: Supplementary Figure 3 — Transmission dynamics between discrete states. (A) Inferred mean transmission matrix of H5N8 viruses between the provinces. (B) Inferred mean transmission matrix of H5N8 viruses between the hosts. [file Image_3.JPEG]
