# Supplementary material for: Evolution, Transmission, and Pathogenicity of High Pathogenicity Avian Influenza Virus A (H5N8) Clade 2.3.4.4, South Korea, 2014–2016
Source: Front Vet Sci. 2022 Jun 21;9:906944. doi: 10.3389/fvets.2022.906944 (PMC9253604; doi:10.3389/fvets.2022.906944)
Supplement: Supplementary Table 1 — Amino acid differences between subgroups and proportion of H1731 mutation having a significance in Shannon entropy. [file Table_1.DOCX]

| Supplementary table 1. Amino acid differences between subgroups and proportion of H1731 mutation having a significance in Shannon entropy | | | | | | | | | | | | | | | | | |
| --- | --- | --- | --- | --- | --- | --- | --- | --- | --- | --- | --- | --- | --- | --- | --- | --- | --- |
|  | |  | C0 (n = 276) | |  | C1 (n=101) | |  | | | C2 (n=93) | |  | | C4 (n=10) | | |
| Gene | Amino acid  change |  | Residue  (No. of strains) | %  of mutation |  | Residue  (No. of strains) | %  of mutation | |  | Residue  (No. of strains) | | %  of mutation |  | Residue  (No. of strains) | | %  of mutation |  |
| PB2 | T184A |  | T(227), A(49) | 17.8 |  | T(7), A(94) | 93.1 | |  | T(93), A(0) | | 0.0 |  | T(10), A(0) | | 0.0 |  |
|  | A442S |  | A(276), S(0) | 0.0 |  | A(91), S(10) | 9.9 | |  | A(93), S(0) | | 0.0 |  | A(10), S(0) | | 0.0 |  |
|  | D678G |  | D(276), G(0) | 0.0 |  | D(0), G(101) | 100.0 | |  | D(93), G(0) | | 0.0 |  | D(10), G(0) | | 0.0 |  |
| PB1 | V219I |  | V(251), I(25) | 9.1 |  | V(1), I(100) | 99.0 | |  | V(83), I(10) | | 10.8 |  | V(10), I(0) | | 0.0 |  |
| PA | V323I |  | V(241), I(35) | 12.7 |  | V(0), I(101) | 100.0 | |  | V(93), I(0) | | 0.0 |  | V(10), I(0) | | 0.0 |  |
|  | V327G |  | V(276), G(0) | 0.0 |  | V(91), G(10) | 9.9 | |  | V(93), G(0) | | 0.0 |  | V(10), G(0) | | 0.0 |  |
|  | T618K |  | T(276), K(0) | 0.0 |  | T(1), K(100) | 99.0 | |  | T(93), K(0) | | 0.0 |  | T(10), K(0) | | 0.0 |  |
| HA | S141P |  | S(276), P(0) | 0.0 |  | S(6), P(95) | 94.1 | |  | S(93), P(0) | | 0.0 |  | S(10), P(0) | | 0.0 |  |
|  | S163N |  | S(237), N(39) | 14.1 |  | S(0), N(101) | 100.0 | |  | S(93), N(0) | | 0.0 |  | S(10), N(0) | | 0.0 |  |
|  | D376N |  | D(241), N(35) | 12.7 |  | D(0), N(101) | 100.0 | |  | D(93), N(0) | | 0.0 |  | D(10), N(0) | | 0.0 |  |
|  | Q455K |  | Q(276), K(0) | 0.0 |  | Q(0), K(101) | 100.0 | |  | Q(93), K(0) | | 0.0 |  | Q(10), K(0) | | 0.0 |  |
|  | A528V |  | A(242), V(34) | 12.3 |  | A(0), V(101) | 100.0 | |  | A(92), V(1) | | 1.1 |  | A(10), V(0) | | 0.0 |  |
| NA | L23I |  | L(276), I(0) | 0.0 |  | L(0), I(101) | 100.0 | |  | L(93), I(0) | | 0.0 |  | L(10), I(0) | | 0.0 |  |
|  | S69N |  | S(274), N(2) | 0.7 |  | S(0), N(101) | 100.0 | |  | S(93), N(0) | | 0.0 |  | S(10), N(0) | | 0.0 |  |
|  | T329I |  | T(266), I(10) | 3.6 |  | T(0), I(101) | 100.0 | |  | T(92), I(1) | | 1.1 |  | T(10), I(0) | | 0.0 |  |
| NS1 | N80S |  | N(266), S(10) | 3.6 |  | N(0), S(101) | 100.0 | |  | N(93), S(0) | | 0.0 |  | N(10), S(0) | | 0.0 |  |
|  | D171E |  | D(275), E(1) | 0.4 |  | D(8), E(93) | 92.1 | |  | D(93), E(0) | | 0.0 |  | D(10), E(0) | | 0.0 |  |
| NS2 | M14K |  | M(276), K(0) | 0.0 |  | M(8), K(93) | 92.1 | |  | M(93), K(0) | | 0.0 |  | M(10), K(0) | | 0.0 |  |
