# Supplementary material for: Evolution, Transmission, and Pathogenicity of High Pathogenicity Avian Influenza Virus A (H5N8) Clade 2.3.4.4, South Korea, 2014–2016
Source: Front Vet Sci. 2022 Jun 21;9:906944. doi: 10.3389/fvets.2022.906944 (PMC9253604; doi:10.3389/fvets.2022.906944)
Supplement: Supplementary Table 2 — Selective pressure analysis of H5N8 highly pathogenic avian influenza viruses isolated in South Korea during 2014-2016. [file Table_2.docx]

| **Supplementary Table 2. Selective pressure analysis of H5N8 highly pathogenic avian influenza viruses isolated in South Korea during 2014-2016** | | | | | | | | | | | | | | | |
| --- | --- | --- | --- | --- | --- | --- | --- | --- | --- | --- | --- | --- | --- | --- | --- |
| **Group** | **Gene** | **Position** | **FEL** | | **FUBAR** | | **SLAC** | | **MEME** | | **Amino acid sequence** | | | | |
|  |  |  | **beta** | **p-value** | **Prob[α<β]** | **BayesFactor[α<β]** | **dN** | **P [dN/dS > 1]** | **β+** | **p-value** | **Buan2 (C0)** | **H1731 (C1)** | **H2102 (C2)** | **H1924 (C4)** |  |
| C0 | PB2 | 694 |  |  |  |  |  |  | 1320.49 | 0.02 | F | F | F | F |  |
|  | PB1 | 219 |  |  | 0.933 | 27.072 |  |  |  |  | V | I | V | V |  |
|  |  | 757 |  |  | 0.95 | 36.302 |  |  |  |  | K | K | K | K |  |
|  | HA | 181 |  |  | 0.951 | 29.001 |  |  |  |  | S | S | S | P |  |
|  |  | 269 |  |  | 0.947 | 26.507 |  |  |  |  | V | V | V | V |  |
|  | M2 | 11 |  |  | 0.921 | 13.232 |  |  |  |  | T | T | T | T |  |
|  |  | 13 |  |  | 0.919 | 12.922 |  |  |  |  | T | T | T | T |  |
|  | NS1 | 109 |  |  |  |  |  |  | 861.35 | 0.09 | Q | Q | Q | Q |  |
|  |  | 180 | 11.334 | 0.0833 | 0.981 | 61.88 |  |  |  |  | V | V | I | V |  |
| C1 | PB2 | 184 |  |  | 0.959 | 34.928 |  |  |  |  | T | A | T | T |  |
|  |  | 559 |  |  | 0.906 | 14.188 |  |  |  |  | I | I | I | I |  |
|  | PB1 | 219 |  |  | 0.933 | 27.072 |  |  |  |  | V | I | V | V |  |
|  |  | 757 |  |  | 0.95 | 36.302 |  |  |  |  | K | K | K | K |  |
|  | NA | 113 |  |  | 0.909 | 14.083 |  |  |  |  | F | F | F | F |  |
|  | M2 | 13 |  |  | 0.959 | 26.871 |  |  |  |  | T | T | T | T |  |
|  |  | 19 |  |  | 0.964 | 31.46 |  |  |  |  | C | C | C | C |  |
|  | NS1 | 59 |  |  | 0.955 | 24.65 |  |  |  |  | R | R | R | R |  |
| C2 | PB1 | 219 |  |  | 0.933 | 27.072 |  |  |  |  | V | I | V | V |  |
|  |  | 757 |  |  | 0.95 | 36.302 |  |  |  |  | K | K | K | K |  |
|  | NP | 183 |  |  | 0.957 | 33.925 |  |  |  |  | V | V | V | V |  |
|  |  | 353 |  |  | 0.901 | 13.977 |  |  |  |  | V | V | V | V |  |
|  | NA | 369 |  |  | 0.946 | 25.041 |  |  |  |  | S | S | S | S |  |
| C4 | NP | 105 |  |  |  |  | 0.333 | 0 |  |  | M | M | M | M |  |
|  |  | 331 |  |  |  |  | 0.333 | 0 |  |  | M | M | M | M |  |
| α | Mean posterior synonymous substitution rate at a site | | | | | | | | |  |  |  |  |  | |
| β | Mean posterior non-synonymous substitution rate at a site | | | | | | | | |  |  |  |  |  | |
| Prob[α<β] | Posterior probability of positive selection at a site | | | | | | | | |  |  |  |  |  | |
| BayesFactor | Empirical Bayes factor for positive selection at a site | | | | | | | | |  |  |  |  |  | |
| β+ | Non-synonymous substitution rate at a site for the positive/neutral evolution component | | | | | | | | |  |  |  |  |  | |
| dN | Rates of fixation of nonsynonymous substitutions | | | | | | | | |  |  |  |  |  | |
| P [dN/dS > 1] | Positive/Diversifying selection | | | | | | | | |  |  |  |  |  | |
